# USING THE LTSS STATE SCORECARD TO MEASURE PROGRESS ON THE 2022 NATIONAL STRATEGY TO SUPPORT FAMILY CAREGIVERS

**DOI:** 10.1093/geroni/igad104.1645

**Published:** 2023-12-21

**Authors:** Eileen Tell, Pamela Nadash

**Affiliations:** ET Consulting, Belmont, Massachusetts, United States; University of Massachusetts Boston, Boston, Massachusetts, United States

## Abstract

Through the Recognize, Assist, Include, Support, & Engage (RAISE) Family Caregivers Act of 2018, the RAISE Family Caregiver Advisory Council was created. Since 2019, the Council has directed in-depth information-gathering and research efforts to develop recommendations and an implementation plan for addressing our critical family caregiving crisis. The Council recently released the 2022 National Strategy to Support Family Caregivers, which highlights nearly 350 actions the federal government will take to support family caregivers in the coming year and more than 150 actions that can be adopted at other levels of government and across the private sector to build a system to support family caregivers. Integrating measures of improvement with regard to family caregiver support, across the domains identified in the National Strategy into the LTSS State Scorecard will provide a tool by which progress toward the RAISE goals can be measured. Currently, the Scorecard focuses on indicators specific to Supporting Working Family Caregivers. While these are critical, our research team has identified at least 10 additional indicators that could be included and that would address other caregiver support domains such as adequacy of direct care workforce, integration of family caregivers into care plans, direct pay to family caregivers, and more. In this session we will identify the indicators, explain how they relate to RAISE goals and discuss methods for including them in the Scorecard.

